# A genetic polymorphism affects the risk and prognosis of renal cell carcinoma: association with follistatin-like protein 1 expression

**DOI:** 10.1038/srep26689

**Published:** 2016-05-26

**Authors:** Yan Liu, Xue Han, Yongwei Yu, Yibo Ding, Chong Ni, Wenbin Liu, Xiaomei Hou, Zixiong Li, Jianguo Hou, Dan Shen, Jianhua Yin, Hongwei Zhang, Timothy C. Thompson, Xiaojie Tan, Guangwen Cao

**Affiliations:** 1 Department of Epidemiology, Second Military Medical University, Shanghai, China; 2 Department of Chronic Diseases, Center for Diseases Control and Prevention of Yangpu District, Shanghai, China; 3 Department of Pathology, the 1^st^ affiliated hospital, Second Military Medical University, Shanghai, China; 4 Department of Urology, the 1^st^ affiliated hospital, Second Military Medical University, Shanghai, China; 5 Genitourinary Medical Oncology-Research, University of Texas M. D. Anderson Cancer Center, Houston, TX, USA

## Abstract

Few single nucleotide polymorphisms (SNPs) associated with the risk of renal cell carcinoma (RCC) have been identified, yet genetic predisposition contributes significantly to this malignancy. We previously showed that follistatin-like 1 (FSTL1) was significantly down-regulated in clear cell RCC (ccRCC), in particular metastatic ccRCC. In the present study, we systemically investigated the associations of the 6 SNPs within FSTL1-coding genomic region with RCC risk and postoperative prognosis. Age- and gender-matched case-control study (417 *vs* 855) indicated that rs1259293 variant genotype CC was significantly associated with an increased risk of RCC, with an odds ratio of 2.004 (95% confidence internal [CI] = 1.190–3.375). Multivariate Cox regression analysis in 309 of 417 cases showed that rs1259293 genotype (CC *vs* TT + CT) independently predicted an unfavorable prognosis, with a hazard ratio of 2.531 (95% CI = 1.052–6.086). Expression of FSTL1 was significantly higher in adjacent renal tissues than in tumors, and significantly higher in the tissues with rs1259293 TT genotype than in those with rs1259293 TC+CC genotypes. rs1259293 C allele might generate a CTCF binding site that blocks *trans*-activation of FSTL1 expression. Our results indicate that rs1259293 is associated with an increased risk and unfavorable postoperative prognosis of RCC, possibly by down-regulating FSTL1 expression in renal tissues.

Renal cell carcinoma (RCC) is the major cancer type in kidney and accounts for about 3% of all human malignancies, with a male-to-female ratio of approximately 2:1^1^,^2^. It derives from renal tubular epithelial cells. Clear cell RCC (ccRCC) accounts for 80% of RCC. A recent twin study carried out in nordic countries has demonstrated that genetic factors contribute greatly to the occurrence of RCC^3^. Genome-wide association studies (GWAS) in western populations have shown that two loci mapped to endothelial PAS domain protein 1 (*EPAS1*) gene, encoding hypoxia inducible factor (HIF)-2α, on 2p21 (rs11894252 and rs7579899), a locus on 11q13.3 (rs7105934), a locus mapped to *SCARB1*, encoding the scavenger receptor class B, member 1, on 12q24.31 (rs4765623), a locus at a transcriptional enhancer of cyclin D1-encoding (*CCND1*) gene (rs7105934) at 11q13.3, two loci (rs718314 and rs1049380) in the inositol 1,4,5-triphosphate receptor, type 2 (*ITPR2*) gene on 12p11.23, and a locus (rs35252396) located at 8q24.21were significantly associated with RCC susceptibility^4^,^5^,^6^,^7^. However, these GWAS-identified RCC-risk single nucleotide polymorphisms (SNPs) identified in western populations are rarely replicated in Chinese populations^8^,^9^. Interestingly, the RCC-risk polymorphisms in genes important for reductive metabolism of chemical exposure in western populations are also not replicated in Chinese^10^,^11^,^12^,^13^. The genetic polymorphisms and their biological effects on signaling proteins that promote RCC development are largely unknown in Chinese population.

In our previous study investigating global gene expression profiling in RCC cells with different metastatic potentials, we showed that follistatin-like 1 (FSTL1) was significantly down-regulated in metastatic ccRCC compared to primary ccRCC cells; furthermore, the mRNA levels of FSTL1 were also significantly lower in ccRCC tissues than in adjacent renal tissue^14^. FSTL1, located on human chromosome 3, can stimulate cell cycle entry and division of pre-existing cardiomyocytes, thus improving cardiac function^15^. FSTL1 functions in cardio-renal communication. The lack of FSTL1 production by myocytes promotes glomerular and tubulointerstitial damage in the kidney^16^. FSTL1 is locally expressed in the loop of Henle in the kidney, and play a role in protecting the kidney from acute nephrotoxic injury *via* mediating interleukin-1β suppression^17^. The role of FSTL1 in cancer is complex and controversial. In cancer metastatic to bone, FSTL1 can mediate cancer cell invasion^18^. In ovarian and endometrial cancers, FSTL1 functions as a tumor suppressor through pro-apoptotic activities^19^. However, to our knowledge, the role of SNPs that affect FSTL1 expression within the context of malignancy had not been reported.

In the present study, a locus mapped to *FSTL1* was not only proven to be associated with RCC risk, but also predicted postoperative prognosis of RCC. The risk genotype of rs1259293 was significantly correlated to reduced FSTL1 expression in adjacent renal tissues. Our results indicate that variant genotype of rs1259293 facilitates development of RCC by down-regulating FSTL1.

## Results

### Characteristics of study population and SNPs

A total of 417 RCC patients including 368 ccRCC cases and 855 healthy controls were enrolled in this study. Age and gender were matched between 417 cases and 855 controls (Table 1). Of the 417 RCC patients enrolled, 108 including 94 ccRCC patients were lost to follow-up after surgery. There were no statistically significant differences in age, gender, and tumor stage between the 309 patients enrolled and the 108 patients excluded in prognosis analysis (data not shown).

From the HapMap projects (www.hapmap.org), FSTL1 coding region was located at 121595817-121652515 of chromosome 3. On the basis of the information in the HapMap project Chinese Han population database, we selected candidate tagged *FSTL1*-related SNPs at an *r*^2^ threshold of 0.80 and minor allele frequency (MAF) of no less than 20% using the Haploview 4.2 program. Six tagged SNPs (rs1105219, rs1259293, rs1402372, rs2673704, rs11708686, and rs1259339) were selected (Supplementary Fig. 1). Of the 6 SNPs, rs11708686 was located in the 3′UTR and the other 5 SNPs were in the intron regions.

### Association of FSTL1-related SNPs with the risk of RCC

The case-control study was designed to investigate the association of FSTL1-related SNPs with the risk of RCC. Six SNPs (rs1105219, rs1259293, rs1259339, rs1402372, rs2673704, and rs11708686) were genotyped in 417 cases and 855 healthy controls. All the six SNP candidates were conformed to Hardy-Weinberg equilibrium (HWE) in healthy controls (*P* > 0.05). Table 2 presents the genotype distributions of the 6 SNPs in healthy controls and RCC patients including ccRCC patients. Compared to healthy controls, the variant genotype CC of rs1259293 in the intron 2 of FSTL1 coding region was significantly associated with an increased risk of RCC, with an odds ratio (OR) of 2.004 and 95% confidence interval (CI) of 1.190–3.375 (*P* = 0.009). Similarly, the CC genotype of rs1259293 was also significantly associated with an increased risk of ccRCC (OR = 2.014, 95% CI = 1.171–3.463, *P* = 0.011). The other 5 SNPs (rs11708686, rs1105219, rs1259339, rs1402372, and rs2673704) were not significantly related to the risk of RCC or ccRCC.

### FSTL1-related SNPs predicted postoperative prognosis in RCC

**Figure 1:**
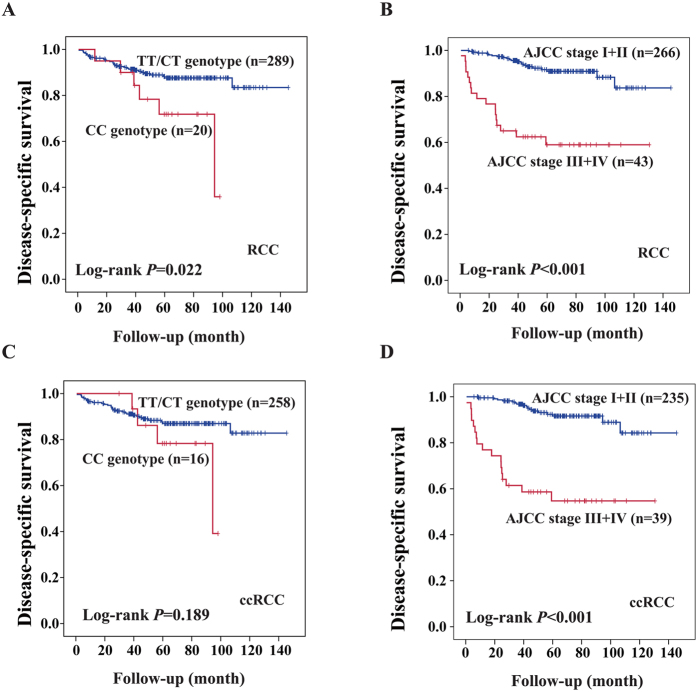
Kaplan-Meier analysis and Log-rank test showed the effect of the factors significantly associated with the risk of renal cell carcinoma (RCC) on predicting postoperative prognosis in RCC. (**A**) Comparison of the CC genotype and the TC and TT genotypes of rs1259293 in predicting disease-specific survival (DSS) in RCC. (**B**) Comparison of advanced AJCC stages (III+IV) and early stages (I+II) in predicting DSS in RCC. (**C**) Comparison of the CC genotype and the TC and TT genotypes of rs1259293 in predicting DSS in clear cell RCC (ccRCC). (**D**) Comparison of advanced AJCC stages (III+IV) and early stages (I+II) in predicting DSS in ccRCC.

Of the 417 RCC patients, 309 RCC patients including 274 ccRCC patients were successfully followed up after surgery. Up to the last follow-up date on January 30, 2015, 38 patients including 34 ccRCC patients died of RCC following relapse. The association of each SNP with disease-specific survival (DSS) of RCC was evaluated using Kaplan-Meier analysis. It was found that the CC genotype of rs1259293 significantly predicted an unfavorable postoperative prognosis in RCC (Log-rank test, *P* = 0.022, Fig. 1A) and that none of other 5 SNPs were statistically associated with postoperative prognosis in RCC (data not shown). The CC genotype of rs1259293 did not significantly predict prognosis in ccRCC patients (Log-rank test, *P* = 0.189, Fig. 1C), however, the average survival durations of ccRCC patients with TT/CT genotypes and CC genotype of rs1259293 were 128.517 ± 3.074 and 85.455 ± 5.681 months, respectively. Advanced AJCC stages (III-IV *vs* I-II) also predicted an unfavorable postoperative prognosis in RCC (Log-rank test, *P* < 0.001, Fig. 1B); this effect was replicated in ccRCC (Log-rank test, *P* < 0.001, Fig. 1D). No significant differences in age, gender, and AJCC stage were found between RCC patients and ccRCC patients with TT/CT genotypes or CC genotypes. Multivariate Cox regression analysis including age, gender, AJCC stage, and rs1259293 genotype showed that AJCC stage (III- IV *vs* I-II) and rs1259293 genotype (CC *vs* TT + CT) conferred an unfavorable postoperative prognosis in RCC independently (Table 3).

### Association of rs1259293 genotypes with FSTL1 expression in renal tissues

**Figure 2:**
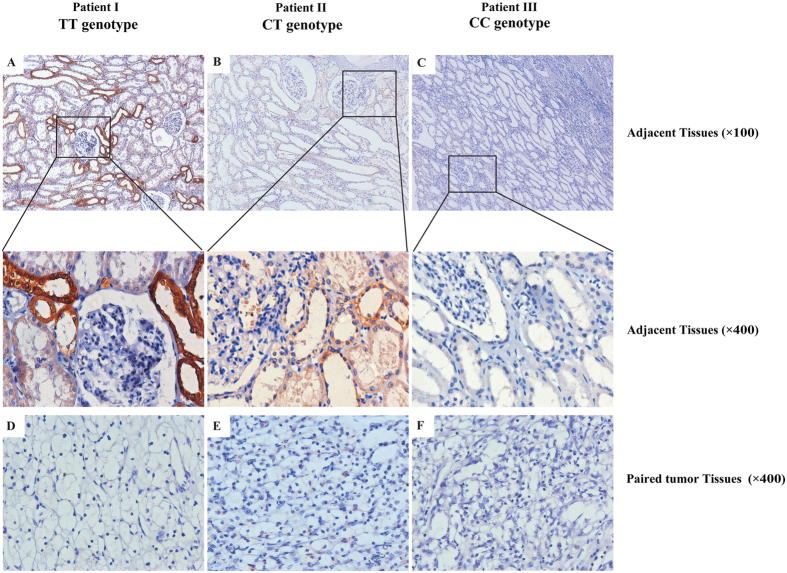
Expression pattern of FSTL1 in adjacent normal renal tissues and paired ccRCC tissues using immunohistochemistry. (**A**) Representative FSTL1 immunostaining in adjacent normal renal tissues of the patient with rs1259293 TT genotype. (**B**) Representative FSTL1 immunostaining in adjacent normal renal tissues of the patient with rs1259293 CT genotype. (**C**) Representative FSTL1 immunostaining in adjacent normal renal tissues of the patient with rs1259293 CC genotype. (**D**) Representative FSTL1 immunostaining in paired tumor tissues of the patient with rs1259293 TT genotype. (**E**) Representative FSTL1 immunostaining in paired tumor tissues of the patient with rs1259293 CT genotype. (**F**) Representative FSTL1 immunostaining in paired tumor tissues of the patient with rs1259293 CC genotype.

**Figure 3:**
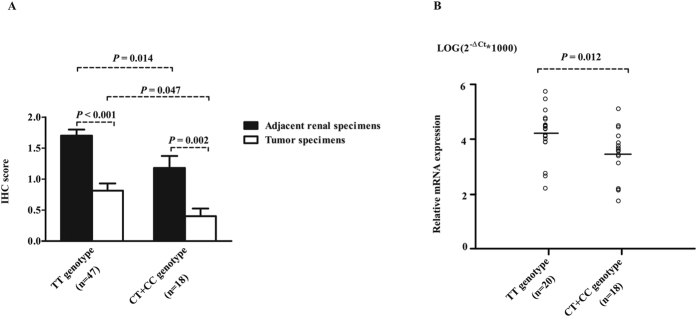
Comparison of FSTL1 expression level in adjacent renal tissues and paired tumor tissues of the ccRCC patients. (**A**) IHC scores of FSTL1 immunostaining in tumors and adjacent renal tissues of patients with rs1259293 TT genotype (n = 47) and those with rs1259293 TC/CC genotypes (n = 18). (**B**) The levels of FSTL1 mRNA in adjacent renal tissues of 20 newly enrolled patients from the 47 patients with rs1259293 TT genotype and all the 18 patients with rs1259293 TC/CC genotypes. IHC, immunohistochemistry.

To determine whether rs1259293 genotype was related to the expression level of FSTL1 in the kidney, we examined FSTL1 expression in formalin-fixed paraffin-embedded (FFPE) specimens of ccRCC and the paired adjacent pathologically normal renal tissues from 65 patients using immunohistochemistry (IHC). In adjacent pathological normal renal tissues, FSTL1 immunostaining was selectively positive in the cytoplasm of the loop of Henle near distal convoluted tubules; moreover, the expression of FSTL1 was higher in renal tissues with rs1259293 TT genotype than in those with rs1259293 CT genotype and FSTL1 was almost negative in those with the CC genotype (Fig. 2). Spearman co-efficient test showed that rs1259293 TT genotypes (*vs* CT + CC genotype) was significantly correlated to higher IHC score of FSTL1 expression in adjacent renal tissues (*P* = 0.041) and but not significantly correlated to FSTL1 expression in tumors (Table 4). Furthermore, rs1259293 TT genotypes (*vs* CT + CC genotype) was significantly correlated to higher FSTL1 expression if the IHC scores of ccRCC and the paired adjacent normal renal specimens were combined as a value of a patient (*P* = 0.007). In RCC patients with rs1259293 TT genotype, IHC score of FSTL1 was significantly higher in adjacent normal renal tissues than in tumor tissues. As sample size of RCC patients with rs1259293 CC genotype was small (n = 5), we combined RCC patients with rs1259293 CC genotype and those with rs1259293 TC genotype. IHC score of FSTL1 was significantly higher in adjacent normal renal tissues than in tumor tissues in the RCC patients with rs1259293 CT + CC genotypes. The IHC score was also significantly higher in adjacent normal renal tissues of patients with rs1259293 TT genotype than in those with rs1259293 CT + CC genotypes; the same was true in tumor tissues. The expression level of FSTL1 mRNA was significantly higher in adjacent renal tissues with TT genotype than in those with CT + CC genotypes by detecting the corresponding FFPE specimens, which was accordance with the IHC results. These data were presented in Fig. 3.

## Discussion

In this study, we showed that a genetic polymorphism at rs1259293, a locus that has never been linked to any disease so far, predisposed RCC risk. Compared to the major genotype TT, the variant genotype CC of rs1259293 was significantly associated with an increased risk of RCC; furthermore, the CC genotype (*vs* TT + CT) of rs1259293 predicted an unfavorable postoperative prognosis in RCC independently. Thus, the abundance of rs1259293 CC genotype can predispose the susceptibility and unfavorable prognoses of RCC. rs1259293 CC genotype predisposed the susceptibility of ccRCC; however, it did not significantly predict postoperative prognosis in ccRCC. This result is mainly due to small sample size and the long-term survival nature of ccRCC. In the 309 RCC patients involved in the survival study, 20 died of this malignancy; whereas 16 of 274 ccRCC patients died of this malignancy. Thus, 20.0% of deaths (4/20) were taken off from the subsequent prognostic analysis in ccRCC patients, which undoubtedly affected the statistical power. The average survival durations of ccRCC patients with TT/CT genotypes and CC genotype of rs1259293 were 128.517 ± 3.074 and 85.455 ± 5.681 months, respectively. No significant differences in age, gender, and AJCC stage were found between RCC and ccRCC patients with TT/CT genotypes or CC genotypes. The difference in the postoperative prognosis should be mainly contributed by rs1259293 genotypes. Although rs1259293 CC genotype did not predict an unfavorable postoperative prognosis in ccRCC statistically, the trend was quite apparent (Fig. 1C). Thus, we believe that rs1259293 is an important genetic risk factor of RCC on the basis of our results in a case-control study Chinese RCC patients because it is also an independent prognostic factor for RCC in the cohort study. Allelic frequencies of SNPs differ among populations with different racial backgrounds. We checked allelic frequencies of rs1259293 in African American, Caucasian American, and Chinese Han population in the HapMap project (www.hapmap.org). It was found that the frequency of CC genotype at rs1259293 was 5.3% in African American, 53% in Caucasian American, and 6.7% in Chinese Han population. Interestingly, the incidence of RCC was 12.5/10^5^ in male Caucasian American and 6.7/10^5^ in female Caucasian American; whereas the incidence of RCC was 5.5/10^5^ in Chinese men and 2.7/10^5^ in Chinese women^20^. Higher CC frequency at rs1259293 is associated with higher RCC incidence in Caucasian than in Chinese, suggesting that the CC genotype at rs1259293 plays a critical role in renal carcinogenesis. However, this relationship might be not evident between African American and Chinese, because the incidence of RCC was 15.2/10^5^ in male African American and 7.3/10^5^ in female African American^20^. This is possibly because other strong factors such as chronic kidney disease overwhelm the effect of rs1259293 CC genotype. We also found that urolithiasis independently increased the risk of RCC in Chinese^13^. Having a history of chronic kidney disease is associated with an almost 3-fold increased risk of RCC and this association is strongest among black people (OR = 10.4 [95% CI = 6.0–17.9])^21^. Nevertheless, the association of rs1259293 with RCC risk and RCC prognosis should be validated in populations with different racial background.

To elucidate the mechanisms by which the rs1259293 genotype predisposed the susceptibility and predicted postoperative prognosis in RCC, we investigated the association of the rs1259293 genotype with FSTL1 expression in tumors and adjacent renal tissues. We found that the IHC score of FSTL1 expression in adjacent renal tissues reduced consecutively from the cortex with rs1259293 TT genotype, those with rs1259293 TC genotype, and those with rs1259293 CC genotype (Fig. 2). The C allele at rs1259293 was proven to be significantly correlated to low IHC score of FSTL1 in adjacent normal renal tissues (Table 4). Expression of FSTL1 was significantly higher in adjacent normal renal tissues than in paired tumor tissues and significantly higher in the tumor or paired adjacent renal tissues of RCC patients with rs1259293 TT genotype than in those with rs1259293 CT + CC genotypes at the protein level (Fig. 3A). Interestingly, the level of FSTL1 mRNA was also significantly higher in adjacent renal tissues with rs1259293 TT genotype than in those with rs1259293 TC+CC genotypes (Fig. 3B). These results indicate that FSTL1 might be a tumor suppressor in RCC while rs1259293 C allele suppresses the transcription of FSTL1 gene. Furthermore, we searched ensemble database at http://asia.ensembl.org/Homo_sapiens/Variation/ and found that rs1259293 C allele, rather than the T allele, generated a CCCTC-binding factor (CTCF)-binding site. CTCF that can bind many enhancer-blocking elements is the only known major insulator-binding protein in the vertebrates and plays important roles in the barrier activity of insulators^22^. Reduced CTCF binding is associated with loss of insulation between topological domains and aberrant gene activation^23^. A genetic variant rs60507107 in the binding site of CTCF was found to be associated with an increased risk of lung cancer^24^. Enhanced binding of CTCF to the sequence with the C allele of rs1259293 may serve as an insulator that blocks active *trans*-activation of FSTL1 promoter and/or enhancer, thus reducing FSTL1 expression. Based on this straightforward mechanistic rationale and the results of our study, it is reasonable to speculate that FSTL1 is a tumor suppressor in RCC, and rs1259293 CC genotypes contribute to low FSTL1 expression, which therefore predicts a poor prognosis. The role of FSTL1 in RCC and its associations with RCC risk factors such as hypertension, obesity, and diabetes^13^,^25^,^26^ and RCC protective factors such as the use of statins or vitamin C^27^,^28^ merit extensive investigation.

In conclusion, the present study systemically investigated the associations of the 6 FSTL1-related SNPs with RCC risk and postoperative prognosis, and identified a new locus rs1259293 whose variant genotype significantly increased RCC risk and predicted an unfavorable postoperative prognosis. Expression of FSTL1 was significantly higher in adjacent normal renal tissues than in paired tumor tissues and significantly higher in the tumor or paired adjacent renal tissues of RCC patients with rs1259293 TT genotype than in those with rs1259293 CT + CC genotype. rs1259293 C allele may generate a CTCF-binding site increasing the binding of CTCF as insulator that blocks active *trans*-activation of FSTL1 enhancer, thus repressing the expression of FSTL1. Further large-scale, well-designed, different racial population-based studies are warranted to elucidate the impact of rs1259293 on RCC risk and postoperative prognosis.

## Methods

### Study population

Peripheral blood samples, tumor tissues and paired adjacent renal tissues were collected from the patients who received curative nephrectomy and were pathologically confirmed RCC at the 1st affiliated hospital of Second Military Medical University from Dec 1998 to Nov 2011. The histology for each case was re-confirmed by at least two pathologists. Healthy controls were recruited from Healthy Examination Center of the 1st affiliated hospital for individuals receiving routine physical examinations between May 2006 and November 2011. All healthy controls had no medical history of genetic diseases, chronic renal diseases or cancer. Demographic information was collected using standard questionnaire by checking their medical records. All participants were Han Chinese. This study was approved by the institutional review board of Second Military Medical University. The methods were carried out in accordance with the approved guidelines. The study subjects provided written informed consents.

### Selection of SNPs

Online Haploview 4.2 software was applied to retrieve the FSTL1-related SNPs from Chinese Han population database in HapMap project (http://hapmap.ncbi.nlm.nih.gov/). Candidate tagged SNPs were selected based on following restrictions: (1) they were located at chromsome 3, 121595000–121653000 region; (2) each had minor allele frequency (MAF) of >20% in Chinese Han population, rs1259339 with MAF of 19% was manually added; (3) each had an r^2^ of >0.80. Thus, 6 tagged SNPs (rs1105219, rs1259293, rs1402372, rs2673704, rs11708686, and rs1259339) were selected.

### Case-control study

The minimum sample size of case group was 376, which was determined by the formula of 
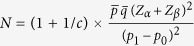
 (α = 0.05, β = 0.10, *c* = 2, *p*_0_ = 0.2, OR = 1.6, 
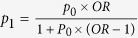
, 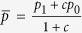, 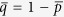). Thus, 417 RCC cases and 855 healthy controls met the need of the minimum sample size in this study. Cases and controls were age- and gender-matched on frequency. Of 417 RCC cases, 368 were diagnosed as ccRCC. Genomic DNA was isolated from peripheral blood of cases and healthy controls, and then subjected for genotyping using the fluorescent-probe real-time quantitative PCR assay as previously described^29^,^30^. The sequences of the primers and probes are listed in Supplementary Table 1. For quality control purposes, two blank controls were included in each 96-well plate, and more than 5% of samples were randomly selected for duplication, yielding a 100% concordance.

### Cohort study

All of the cases genotyped in case-control study were invited to participate in cohort study. The patients were followed up by phone or face-to-face interview every 6 months according to our standard epidemiologic procedure. We excluded the RCC patients who lost contact information and refused to adhere to the follow-up study requirements. The last follow up date was Jan 30, 2015, with a median follow-up duration of 56.56 months (interquartile range: 38.06–81.38 months). Death from RCC relapse was defined as an event. Patients alive at the last follow-up and died of conditions unrelated to RCC were censored. DSS was measured in months from the date of receiving surgery to the date that patient died of ccRCC.

### IHC

Full sections of FFPE specimens of ccRCC and the paired adjacent normal renal tissues were processed using standard techniques. Antigen retrieval was conducted with in 0.01M Tris-EDTA buffer (pH = 8.0) for 25 min at 100 °C in an electric cooker. Sections were blocked and incubated overnight with anti-FSTL1 (C-term) (1:50 dilution; Abgent, AP10534b, San Diego, CA) overnight at 4 °C. FSTL1 was detected using DAB staining system. Staining evaluation was performed independently by three investigators (Tan XJ, Liu Y, and Yu YW) who were blind to the clinicopathological characteristics and outcome of the patients as previously described^30^. Briefly, an immunoreactive score was ranked by negative (−), slightly positive (+), moderately positive (++) and strongly positive (+++) according to the extent and intensity of staining. Furthermore, we accessed each pathological site of the adjacent normal renal tissues independently, and then summed up as the score of adjacent normal renal tissues. There was a close agreement on immunoreactive scores (90%) between two investigators. In cases of disagreement, consensus was obtained after discussion.

### quantitative RT-PCR

The total of 38 FFPE specimens of adjacent renal tissues (20 with TT genotype; 18 with CT + CC genotypes) were involved in this assay. Total RNAs were isolated using RNeasy FFPE kit (Qiagen, 73504, Stockach, Germany) and reverse transcribed to cDNA, and subjected for quantitative RT-PCR as previously described^30^. The primers of *FSTL1* were sense AAATGCAGCTCCCTGTCCAA and reverse ACTCTTGCCCTCCTCCCATAG. The primers of *GAPDH* were sense TGACTTCAACAGCGACACCCA and reverse CACCCTGTTGCTGTAGCCAAA. The relative normalized quantity of *FSTL1* expression was calculated as previously described^31^.

### Statistical analysis

HWE was examined by using online analytical tools (http://ihg.gsf.de). Demographic characteristics between cases and controls were analyzed using Chi-square test. Differences in continuous variables were tested by Student *t* test. Unconditional logistic regression model was conducted to calculate odds ratios (ORs) and their 95% confidence internals (CIs) of the association between the SNPs and RCC risk, adjusting for age and gender. Non-parametric analyses of Spearman correlation test was used to assess the correlation of rs1259293 genotypes to FSTL1 expression. For postoperative prognosis analysis, DSSs and their 95% CIs were estimated by the Kaplan-Meier method. The log-rank test was applied to compare DSS between groups. All statistical tests were two-sided and conducted using Statistical Program for Social Sciences (SPSS 16.0, Chicago, IL, USA). A *P*-value of <0.05 was considered as statistically significant.

## Additional Information

**How to cite this article**: Liu, Y. *et al.* A genetic polymorphism affects the risk and prognosis of renal cell carcinoma: association with follistatin-like protein 1 expression. *Sci. Rep.* **6**, 26689; doi: 10.1038/srep26689 (2016).

## Supplementary Material

Supplementary Information

## Figures and Tables

**Table 1:** Demographic and pathological characteristics of the study subjects.

Characteristic	Case-control study			Survival analysis
	RCC cases (%) N = 417	Controls (%) N = 855	*P*	RCC cases (%) N = 309
Age (years)				
Mean ± SD	56.82 ± 12.85	58.44 ± 15.24	0.543	56.88 ± 13.06
≤60	258 (61.87)	544 (63.63)	–	190 (61.49)
>60	159 (38.13)	311 (36.37)	–	119 (38.51)
Gender				
Male	281 (67.39)	609 (71.23)	0.161	216 (69.90)
Female	136 (32.61)	246 (28.77)	–	93 (30.10)
Histology				
Clear cell	368 (88.25)	–	–	274 (88.67)
Papillary	13 (3.12)	–	–	11 (3.56)
Chromophobe	10 (2.40)	–	–	8 (2.59)
Unclassified	26 (6.24)	–	–	16 (5.18)
AJCC stage				
I–II	373 (89.45)	–	–	266 (86.08)
III–IV	44 (10.55)	–	–	43 (13.92)

Abbreviation: AJCC = American Joint Committee on Cancer; RCC = renal cell carcinoma.

**Table 2:** The associations of FSTL1 polymorphisms with the risk of RCC and ccRCC.

Genotype	RCC cases No (%)	ccRCC cases No (%)	Controls No (%)	Adjusted OR (95% CI)^a^	*P*-value^a^	Adjusted OR (95% CI)^b^	*P*-value^b^
rs11708686 (RCC n = 412, ccRCC n = 364, Control n = 836) H-W *P* = 0.415							
GG	70 (16.99)	64 (17.58)	154 (18.42)	1.000 (Reference)	–	–	–
AG	203 (49.27)	178 (48.90)	423 (50.60)	1.069 (0.769–1.486)	0.691	1.027 (0.730–1.445)	0.877
AA	139 (33.74)	122 (33.52)	259 (30.98)	1.181 (0.830–1.682)	0.355	1.136 (0.788–1.638)	0.494
AG+AA	342 (83.01)	300 (82.42)	682 (81.58)	1.115 (0.816–1.524)	0.493	1.067 (0.773–1.474)	0.692
G allele	343 (41.63)	306 (42.00)	731 (43.72)	1.000 (Reference)	–	–	–
A allele	481 (58.37)	422 (58.00)	941 (56.28)	1.095 (0.925–1.298)	0.291	1.077 (0.903–1.286)	0.408
rs1105219 (RCC n = 397, ccRCC n = 352, Control n = 829) H-W *P* = 0.262							
AA	87 (21.91)	77 (21.88)	168 (20.27)	1.000 (Reference)	–	–	–
AG	205 (51.64)	183 (51.99)	428 (51.63)	0.933 (0.685–1.270)	0.658	0.935 (0.679–1.289)	0.682
GG	105 (26.45)	92 (26.14)	233 (28.11)	0.860 (0.607–1.218)	0.395	0.854 (0.594–1.229)	0.395
AG+GG	310 (78.09)	275 (78.13)	661 (79.73)	0.906 (0.676–1.214)	0.508	0.906 (0.669–1.229)	0.527
A allele	379 (47.73)	337 (47.87)	764 (46.08)	1.000 (Reference)	–	–	–
G allele	415 (52.27)	367 (52.13)	894 (53.92)	0.930 (0.785–1.103)	0.405	0.927 (0.777–1.106)	0.400
rs2673704 (RCC n = 412, ccRCC n = 362, Control n = 851) H-W *P* = 0.170							
TT	21 (5.10)	18 (4.97)	34 (4.00)	1.000 (Reference)	–	–	–
CT	135 (32. 77)	124 (34.25)	241 (28.32)	0.915 (0.509–1.644)	0.765	0.982 (0.531–1.815)	0.953
CC	256 (62.14)	220 (60.77)	576 (67.69)	0.705 (0.400–1.243)	0.228	0.710 (0.392–1.288)	0.260
CT + CC	391 (94.90)	344 (95.03)	817 (96.00)	0.770 (0.440–1.347)	0.360	0.795 (0.442–1.429)	0.443
T allele	177 (21.48)	160 (22.10)	309 (18.16)	1.000 (Reference)	–	–	–
C allele	647 (78.52)	564 (77.90)	1393 (81.84)	0.810 (0.658–0.997)	0.046	0.783 (0.632–0.971)	0.026
rs1259293 (RCC n = 417, ccRCC n = 368, Control n = 855) H-W *P* = 0.124							
TT	247 (59.23)	212 (57.61)	515 (60.23)	1.000 (Reference)	–	–	–
CT	140 (33.57)	130 (35.33)	307 (35.91)	0.946 (0.736–1.217)	0.666	1.023 (0.788–1.327)	0.864
CC	30 (7.19)	26 (7.07)	33 (3.86)	**2.004 (1.190**–**3.375)**	**0.009**	**2.014 (1.171–3.463)**	**0.011**
CT + CC	170 (40.77)	156 (42.39)	340 (39.77)	1.042 (0.820–1.323)	0.737	1.112 (0.868–1.426)	0.400
T allele	634 (76.02)	554 (75.27)	1337 (78.19)	1.000 (Reference)	–	–	–
C allele	200 (23.98)	182 (24.73)	373 (21.81)	1.134 (0.932–1.380)	0.209	1.179 (0.962–1.445)	0.112
rs1402372 (RCC n = 386, ccRCC n = 344, Control n = 835) H-W *P* = 0.744							
CC	58 (15.03)	52 (15.12)	114 (13.65)	1.000 (Reference)	–	–	–
AC	172 (44.56)	157 (45.64)	395 (47.31)	0.868 (0.603–1.251)	0.449	0.889 (0.609–1.298)	0.543
AA	156 (40.41)	135 (39.24)	326 (39.04)	0.963 (0.664–1.397)	0.844	0.928 (0.630–1.366)	0.704
AC+AA	328 (84.97)	292 (84.88)	721 (86.35)	0.909 (0.645–1.282)	0.586	0.901 (0.631–1.287)	0.567
C allele	288 (37.31)	261 (37.94)	623 (37.31)	1.000 (Reference)	–	–	–
A allele	484 (62.69)	427 (62.06)	1047 (62.69)	1.004 (0.842–1.199)	0.961	0.979 (0.815–1.176)	0.820
rs1259339 (RCC n = 386, ccRCC n = 340, Control n = 789) H-W *P* = 0.610							
CC	21 (5.44)	18 (5.29)	34 (4.31)	1.000 (Reference)	–	–	–
CT	131 (33.94)	109 (32.06)	271 (34.35)	0.788 (0.440–1.412)	0.423	0.762 (0.412–1.407)	0.385
TT	234 (60.62)	213 (62.65)	484 (61.34)	0.794 (0.450–1.403)	0.427	0.842 (0.464–1.529)	0.573
CT + TT	365 (94.56)	322 (94.71)	755 (95.69)	0.792 (0.452–1.386)	0.413	0.813 (0.452–1.463)	0.489
C allele	173 (22.41)	145 (21.32)	339 (21.48)	1.000 (Reference)	–	–	–
A allele	599 (77.59)	535 (78.68)	1239 (78.52)	0.950 (0.771–1.170)	0.627	1.010 (0.810–1.258)	0.932

^a^RCC patients *vs* healthy controls.

^b^ccRCC patients *vs* healthy controls.

Abbreviations: FSTL1 = follistatin-like 1; RCC = renal cell carcinoma; ccRCC = clear cell renal cell carcinoma; CI = confidence interval. OR = odds ratio; H-W = Hardy-Weinberg.

**Table 3:** Factors significantly predicted disease-specific survival in multivariate Cox proportional hazards model in 309 RCC patients.

Variables	HR (95% CI)	*P*
Age	1.031 (1.004, 1.058)	0.024
Gender (male *vs* female)	1.308 (0.641, 2.670)	0.461
AJCC stage (III–IV *vs* I–II)	5.907 (3.045, 11.460)	<0.001
rs1259293 genotype (CC *vs* TT + CT)	2.531 (1.052, 6.086)	0.038

Abbreviation: AJCC = American Joint Committee on Cancer; FSTL1 = follistatin-like 1; HR = hazard ratio; RCC = renal cell carcinoma.

**Table 4:** The correlation between rs1259293 genotypes and FSTL1 expression in ccRCC tissues and paired adjacent normal tissues from 65 ccRCC patients using spearman association test.

Paired samples	rs1259293 genotypes	IHC scores of FSTL1 No (%)		*P*	r_s_
		−/+	++/+++		
Adjacent normal tissues	TT	18 (27.7)	29 (44.6)	0.041	−0.255
	CT + CC	12 (18.5)	6 (9.2)	–	–
ccRCC tissues	TT	38 (58.5)	9 (13.8)	0.179	−0.169
	CT + CC	17 (26.2)	1 (1.5)	–	–
Adjacent normal tissues and ccRCC tissues	TT	10 (15.4)	37 (56.9)	0.007	−0.332
	CT + CC	10 (15.4)	8 (12.3)	–	–

Abbreviations: FSTL1 = follistatin-like 1; IHC = Immunohistochemistry; ccRCC = clear cell renal cell carcinoma.
