# Supplementary material for: A genetic polymorphism affects the risk and prognosis of renal cell carcinoma: association with follistatin-like protein 1 expression
Source: Sci Rep. 2016 May 26;6:26689. doi: 10.1038/srep26689 (PMC4880907; doi:10.1038/srep26689)
Supplement: Supplementary Information [file srep26689-s1.pdf]

Supplementary materials

A genetic polymorphism affects the risk and prognosis of renal cell carcinoma: association with follistatin-like protein 1 expression

Yan Liu, Xue Han, Yongwei Yu, Yibo Ding, Chong Ni, Wenbin Liu, Xiaomei Hou, Zixiong Li, Jianguo Hou, Dan Shen, Jianhua Yin, Hongwei Zhang, Timothy C. Thompson, Xiaojie Tan, & Guangwen Cao

Supplementary Figure 1.

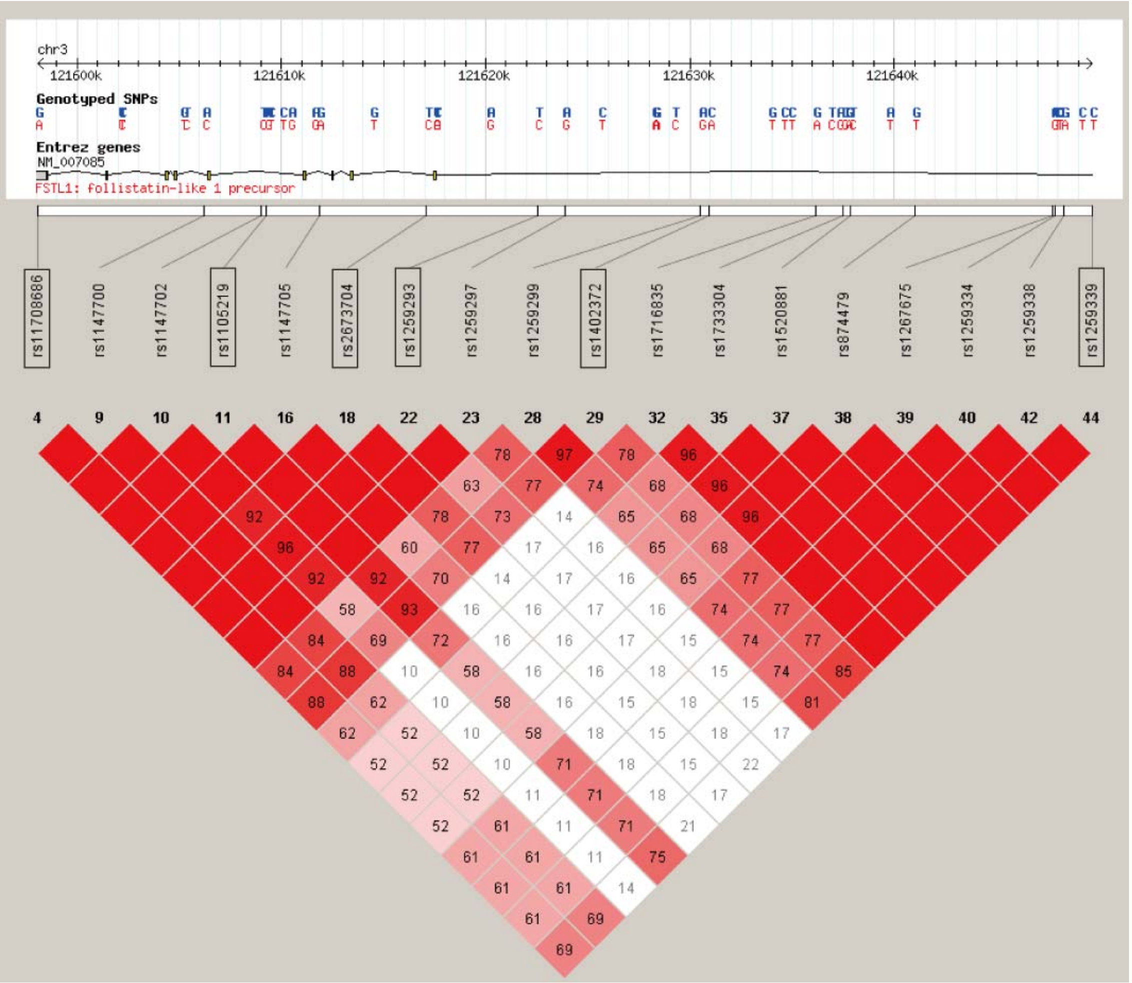

Supplementary Figure 1. Haploview linkage disequilibrium plot and identification of haplotype block in the follistatin-like protein 1 (FSTL1) gene. The positions of the SNPs within exons, introns, and 3'-untranslated region of FSTL1 gene are shown above the plot. Pairwise linkage disequilibrium (LD) coefficients D'×100, indicating extent of LD between SNPs, are shown in each square (D′ values of 1.0 are not shown). A standard Haploview color scheme was applied for the LD color display. Higher color intensity of the squares indicates higher LD between SNPs. The inverted black triangle represents a single haplotype block.

Supplementary Table 1. Primers and probes for selected SNP genotyping.

Polymorphisms	Primer sequences (5'-3')	Probe sequences (5'-3')
rs11708686	Forward: GGTCTGTCATGCTGACGGC	FAM-AAGAGGCTGGAAGCAGAGAgCGTTCC-BHQ
	Reverse: CGGAGGAGAGGTTTGGGAA	HEX-AAGAGGCTGGAAGCAGAGAaCGTTC-BHQ
rs2673704	Forward: ATCTTCCCCCATAACCCTTC	FAM-CCAGCGTTAACAATTTGgTACATGTTCCA-BHQ
	Reverse:AGCATATAAACCCTATGCTTATAGTAACATAGA	HEX-CCAGCGTTAACAATTTGaTACATGTTCC-BHQ
rs1259293	Forward: GCAAGCAAATGGAGAAGCAAG	FAM-TTGCAAAACcACCAGTAG-MGB
	Reverse: CAGAGACTCAGATGCACGATCAC	HEX-TTTGCAAAACtACCAGTAGA-MGB
rs1259339	Forward: AATTGAGGCTTCTTGGTGCTTT	FAM-CTTTTCTGCaACCAT-MGB
	Reverse:TTTACTACAGAGGCTCTGGCTTCTT	HEX-TTTTCTGCgACCATAA-MGB
rs1105219	Forward: CAGCATGTAAGACTCTGATAATCATCAA	FAM-TGTTTTCTCCTGTTAGaCA-MGB
	Reverse:AAATCCCCTCTTCCCAGACACT	HEX-CTGTTAGgCACTGCC-MGB
rs1402372	Forward:AAACCTTTTAAACAGACACACTTGGA	FAM-AACAGGcCTAAACTG-MGB
	Reverse: CCACAAATTTTCCCATCCCATA	HEX-AACAGGaCTAAACTGT-MGB

Abbreviation:FSTL1=follistatin-like 1; SNP=single-nucleotide polymorphism; FAM=6-carboxyfluorescein; HEX=6-hexachloro-fluorescein; BHQ=Black Hole Quencher ; MGB=minor groove binder.
